# Prevalence of tuberculosis in pigs slaughtered at two abattoirs in Ethiopia and molecular characterization of *Mycobacterium tuberculosis* isolated from tuberculous-like lesions in pigs

**DOI:** 10.1186/1746-6148-9-97

**Published:** 2013-05-06

**Authors:** Sintayehu Mulugeta Arega, Franz Josef Conraths, Gobena Ameni

**Affiliations:** 1 Department of Veterinary Clinical Medicine, Faculty of Veterinary Medicine, University of Gondar, P. O. Box 196, Gondar, Ethiopia; 2 Friedrich-Loeffler-Institute, Federal Research Institute for Animal Health, Institute of Epidemiology, Seestraße 55, 16868, Wusterhausen, Germany; 3 Aklilu Lemma Institute of Pathobiology, Addis Ababa University, PO Box 1176, Addis Ababa, Ethiopia; 4 Freie Universität Berlin, Koenigsweg 67, Berlin, 14163, Germany

**Keywords:** Abattoir, Ethiopia, Molecular typing, *Mycobacterium tuberculosis*, Pig, *Post-mortem examination*, Tuberculosis

## Abstract

**Background:**

Tuberculosis (TB) is an infectious, granulomatous disease caused by acid-fast bacilli of the genus *Mycobacterium*. The disease affects practically all species of vertebrates. Although mammalian tuberculosis has been nearly controlled in many developed countries, it is still a serious problem in humans and domestic animals including pigs in developing countries. In Ethiopia, the prevalence of TB in pigs is not known. Therefore, this study was designed to estimate the prevalence of TB in pigs in central Ethiopia and to characterize the causative agents using molecular techniques.

**Results:**

The estimated prevalence of TB was 5.8% (49/841). Age and origin of pigs were significantly associated (P<0.001) with the prevalence. In contrast, an association of sex, floor type and water source with the prevalence could not be shown. Culture positivity was confirmed in 30.6% (15/49) of the tuberculous-like lesions. Of the 15 isolates, 12 were acid fast positive while five of the latter were confirmed by multiplex PCR as members of the *M. tuberculosis* complex. Speciation of the five isolates further confirmed that they were *M. tuberculosis*, belonging to SIT1088 (two isolates) and SIT1195 (one isolate). The remaining two isolates belong to an identical spoligotype, the pattern of which was not found in the spoligotype database (SpolDB4).

**Conclusions:**

The isolation of *M. tuberculosis* from pigs suggests a possible risk of transmission between humans and pigs. Hence, establishing feasible control methods is required.

## Background

Tuberculosis (TB) is an infectious, granulomatous disease caused by acid-fast bacilli (AFB) of the genus *Mycobacterium*. The disease affects practically all species of vertebrates [1]. The tubercle bacilli are *Mycobacterium (M.) tuberculosis*, the agent of the disease in primates, *M. bovis* in other mammals and *M. avium* in birds. Host specificity is relative [2].

Pigs are susceptible to all the three types of tubercle bacilli [1,3]. It has been suggested that there is a correlation between the occurrence of TB in pigs and a direct or indirect contact of pigs with tuberculous humans, cattle or birds [4,5]. The presence of TB in pigs in virtually all countries, in which pigs are farmed, has long been reported [4]. In Ethiopia, a retrospective meat inspection data analysis report by Shitaye and co-workers [6] indicated a 0.009% prevalence of TB in pigs slaughtered in Addis Ababa abattoir in the years 1996–2005. This report is the only available information on tuberculosis in pigs in Ethiopia. The mycobacterial species involved were not isolated in the study.

The impact of tuberculosis in swine is complicated. Pig carcasses are condemned due to tuberculous lesions [7]. Losses from test-and-slaughter of cattle for the control of bovine TB caused by dissemination of *M. bovis* by infected pigs [8,9] and losses from destruction of false-positive reactors due to sensitization by mycobacteria species other than tuberculosis [10-12] may be indirect consequences of swine tuberculosis. On top of this, an increasing number of reports of several types of mycobacterial infections among HIV–infected and immunocompromised patients has blamed animals to be the source of infections to humans [13-15]. Studies have shown that pigs are among the possible sources of mycobacterial infections to humans and animals [9,16,17].

Although mammalian tuberculosis has been nearly controlled in humans and domestic animals in many developed countries, it is still a serious problem in developing countries including Ethiopia [18-20]. In Ethiopia, the epidemiology of tuberculosis is barely studied. Particularly in pigs, it is not known at all. The detection, isolation and characterization of the causative agents and investigation of risk factors associated with the disease are important steps in the control of TB. Therefore, this study was designed to estimate the prevalence of TB in pigs in central Ethiopia and to characterize the causative agents using molecular techniques.

## Methods

### Study area

The investigated specimens and epidemiological data from pigs were collected from two abattoirs located in Addis Ababa and Bishoftu. Both cities are located in the central part of Ethiopia. Addis Ababa is the capital and largest city of the country, while Bishoftu is a rapidly growing and industrializing town located 46 km south-east of Addis Ababa. Apart from Addis Ababa and Bishoftu pigs were brought for slaughter to these abattoirs from Special Oromia Zone, Adama and Modjo which are at most within 100km radius from Addis Ababa.

### Sampling and *post-mortem* examination

A total number of 841 pigs slaughtered from March to September 2011 were investigated. Detailed *post-mortem* examinations were conducted on all slaughtered pigs following the procedure described previously [21]. Lymph nodes and suspected organs were incised into slices of approximately 2 mm thickness to facilitate the detection of lesions and inspected for the presence of TB lesions. Tissues from suspected TB lesions were collected into sterile universal bottles filled with 5 ml of 0.9% saline solution for mycobacterial culture. Then, they were transported in an ice box with ice packs until they reached to Aklilu Lemma Institute of Pathobiology (ALIPB) TB laboratory. In the laboratory samples were either processed in the following few days while kept at 4°C until the time of processing or kept refrigerated at −20°C for a maximum of 10 days for later processing.

### Tissue preparation, culturing and identification of *mycobacteria*

Samples were further processed for isolation of mycobacteria in accordance with protocols of the World Organization for Animal Health [22]. In the laboratory, tissue samples were dissected using sterile blades and manually homogenized using a mortar and pestle. This was followed by decontamination by shaking the homogenate in an equal volume of 4% NaOH for 10–15 minutes at room temperature and neutralized with 1% (0.1N) HCl using phenol red as an indicator. Neutralization was achieved when the colour of the solution turned from purple to yellow. The suspension was then centrifuged 3,000 ×g for 15 minutes, the supernatant discarded and sediment was used for AFB staining and culture.

The sediment was cultured onto each slant of two Lowenstein–Jensen (LJ) media one enriched with glycerol and the other with pyruvate. The slants were incubated aerobically at 37°C for a minimum of eight weeks or until macroscopic growth was observed while they were examined on a regular basis for macroscopic growth. Smears from visible growth were prepared for microscopic examination using the Ziehl–Neelsen staining technique to select AFB positive isolates.

### Molecular characterization of mycobacterial isolates

#### Genus typing by Multiplex PCR

Ten AFB positive isolates were subjected to multiplex PCR identification test according to the technique described by Wilton and Cousins [23], which detects and differentiates *M. tuberculosis* complex (MTC) from *M. avium* complex (MAC), *M. intracellularae* and other mycobacterial species. Heat killed AFB positive samples were used as source of DNA template.

In brief, the PCR amplification reaction was performed in a Thermal Cycler (Applied Biosystems; PTC-100™) with 20 μl reaction mixture used for PCR reaction. This total volume consisted of 5 μl of genomic DNA as a template, 8 μl HotStarTaq Master Mix (Qiagen, United Kingdom), 0.3 μl of each of the six different primers used for multiplex PCR and 5.2 μl Qiagen water. The primers for amplification were MYCGEN-F, 5’-AGA GTT TGA TCC TGG CTC AG-3’ (35 ng/μl); MYCGEN-R, 5’- TGC ACA CAG GCC ACA AGG GA-3’ (35 ng/μl); MYCAV-R, 5’-ACC AGA AGA CAT GCG TCT TG-3’ (35 ng/μl); MYCINT-F, 5’ CCT TTA GGC GCA TGA TGT CTT TA 3’ (75 ng/μl); TB1- F, 5’-GAA CAA TCC GGA GTT GAC AA-3’ (20 ng/μl); and TB-1- R, 5’-AGC ACG CTG TCA ATC ATG TA-3’ (20 ng/μl). *M. tuberculosis* strain (H37Rv) and *M. avium* were used as positive controls while Qiagen water served as a negative control. The reaction mixture was then heated to 95°C for 10 minutes. This was followed by 35 cycles of reaction consisting of 95°C for 1 minute for denaturation; 61°C for 0.5 minute for annealing; 72°C for 2 minutes for extension. Finally, the reaction mixture was maintained at 72°C for 10 minutes.

PCR products were electrophoresed in 1.5% agarose gel in 10× TAE running buffer. Ethidium bromide at ratio of 1:10, 100 bp DNA ladder (Promega Cooperation, USA), and Orange 6× loading dye were used in gel electrophoresis. The gel was visualized in a Multi–image™ light cabinet using Alpha Innotech version 1.2.0.1 (Alpha Innotech Corporation). All members of the genus *Mycobacterium* produce a band of 1030 bp and *M. avium* or subspecies such as *M. avium* subsp. *paratuberculosis* produce an extra band of 180 bp, *M. intracellularae* a band of 850 bp while a band of 372 bp can be amplified from members of the MTC.

#### RD4 Deletion typing

RD4 deletion typing was carried out on isolates that showed a band regarded as specific for the MTC by multiplex PCR. For this deletion typing, the procedure of Cadmus *et al.*[24] was followed. Primers that were used include RD4 FlankF 5’-CTC GTC GAA GGC CAC TAA AG-3’, RD4 FlankR 5’-AAG GCG AAC AGA TTC AGC AT-3’, and RD4 Internal 5’-ACA CGC TGG CGA AGT ATA GC-3’ to check for the presence of the RD4 locus. The HotStarTaq Master Mix system from Qiagen was used for PCR with the primers described above. *M. tuberculosis* H37Rv and *M. bovis* were used as positive control while Qiagen water served as a negative control.

A reaction mixture consisting of 10 μl of HotStarTaq Master Mix, 0.3 μl each of the three primers (RD4 FlankR, RD4 FlankF and RD4 Internal), 2 μl DNA template and 7.1 μl H_2_O Qiagen adding up to a final volume of 20 μl was heated in a Thermal Cycler (Applied biosystem; PTC- 100™) to 95°C for 15 minutes. Then, the reaction underwent 35 cycles consisting of de-naturation at 95°C for 1 minute, annealing at 55°C for 1 minute and extension at 72°C for 1 minute. Finally the reaction mixture was maintained at 72°C for 10 minutes.

PCR products were electrophoresed in 1.5% agarose gel in 10× TAE running buffer with ethidium bromide at a ratio of 1:10. A 100 bp DNA ladder (Promega Cooperation, USA) and Orange 6× loading dye were also used for visual tracking of DNA migration during electrophoresis. The gel was visualized in similar way as described above. The presence of RD4 (*M. tuberculosis*, *M. africanum*) gives a product size of 335 bp (RD4 Internal+RD4 FlankR) and in its absence (*M. bovis*), a product of 446 bp (RD4 FlankR+RD4 FlankF) is amplified.

#### RD9 deletion typing

RD9 deletion typing was applied to isolates that showed the presence of RD4 region. For this deletion typing, a procedure described by Cadmus *et al.*[24] was followed. The primers used for RD9 deletion typing were RD9 FlankF, 5’-AAC ACG GTC ACG TTG TCG TG-3’, RD9 FlankR, 5’-CAA ACC AGC AGC TGT CGT TG-3’ and RD9 Internal, 5’**-**TTG CTT CCC CGG TTC GTC TG**-**3’. The mixture was heated in a Thermal Cycler (Applied Biosystems; PTC- 100™) using an initial hot start of 95°C for 15 minutes, followed by 35 cycles of 95°C for 1 minute, 55°C for 2 minute, and 72°C for 1 minute; a final extension step of 72°C for 10 minutes to complete the cycle. Each PCR reaction tube contained 7.1 μl H_2_O Qiagen, 10 μl HotStarTaq Master Mix, 0.3 μl of each of the three primers (1.5 μM final concentration), 2 μl of DNA templates of samples or controls making the total volume 20 μl. *M. tuberculosis* H37Rv and *M. bovis* were used as positive controls while Qiagen water served as a negative control.

PCR product electrophoresis and visualization was performed by similar technique as described above. The presence of RD9 (i.e. *M. tuberculosis*) gives a product size of 396 bp (RD9 FlankF + RD9 Internal) and its absence (*M. africanum*, *M. bovis*) gives a product size of 575 bp (RD9 FlankF+RD9 FlankR).

#### Spoligotyping

Spoligotyping is a PCR-based technique, which exploits the variability of the direct repeat (DR) region, developed to simultaneously detect and type *M. tuberculosis* complex. Spoligotyping was performed as previously described by Kamerbeek *et al.*[25] and according to the spoligotype kit supplier’s instructions (Ocimum Biosolutions Company, Ijsselstein, The Netherlands). The DR region was amplified by PCR using oligonucleotide primers (DRa: 5’ GGT TTT GGG TCT GAC GAC 3’ and DRb: 5’ CCG AGA GGG GAC GGA AAC 3’) derived from the DR sequence. The DRa is biotinylated at the 5’-end. A total volume of 25 μl of the following reaction mixture was used for the PCR: 12.5 μl of HotStarTaq Master Mix (Qiagen: this solution provides a final concentration of 1.5 mM MgCl2 and 200 μM of each deoxynucleotides triphosphates), 2 μl of each primer (20 pmol each), 5 μl suspension of heat-killed cells (approximately 10 to 50 ng), and 3.5 μl distilled water.

The mixture was heated for 15 minutes at 96°C and then subjected to 30 cycles of 1 minute at 96°C, 1 minute at 55°C, and 30 seconds at 72°C. The amplified product was hybridized to a set of 43 immobilized oligonucleotides, each corresponding to one of the unique spacer DNA sequences within the DR locus. After hybridization, the membrane was washed twice for 10 minutes in 2× SSPE (1× SSPE is 0.18 M NaCl, 10 mM NaH_2_PO_4_, and 1 mM EDTA [pH 7.7])/0.5% sodium dodecyl sulphate at 60°C and then incubated in 1:4000 diluted streptavidin peroxidase (Boehringer) for 45 to 60 minutes at 42°C. The membrane was then washed twice for 10 minutes in 2× SSPE-0.5% sodium dodecyl sulphate at 42°C and rinsed with 2× SSPE for 5 minutes at room temperature. Hybridizing DNA was detected by the enhanced chemiluminescence method (Amersham) and by exposure to an X-ray film (Hyperfilm ECL, Amersham) as specified by the manufacturer.

### Statistical analysis

Collected data were double-entered, classified, filtered and coded using Microsoft Excel® 2007 and analysed using the software package STATA 10.1 (StataCorp, Texas, USA). Univariate analysis was performed using the Chi-squared test. Logistic regression was then used to analyse risk factors that were found significantly associated in the univariate analysis with a cut-off P≤ 0.2. Effects were reported as statistically significant if the *P* value was less than 0.05.

## Results

### Husbandry characteristics

A total of 841 pigs were inspected in two abattoirs where used for present study: more than 62% were from Addis Ababa Abattoir and 37% were in Bishoftu Abattoir. The majority (67%) of the pigs were less than one year of age. Pigs were brought for slaughter mainly from Bishoftu (60%) and Addis Ababa and the neighbouring Special Oromia Zone (30%). Eighty eight per cent of the pigs were fed commercial mixed feed, while only 12% fed on swill, poultry offal and litter in addition to grazing on pasture and/or roaming for garbage.

### Prevalence of tuberculosis in pigs

**Figure 1:**
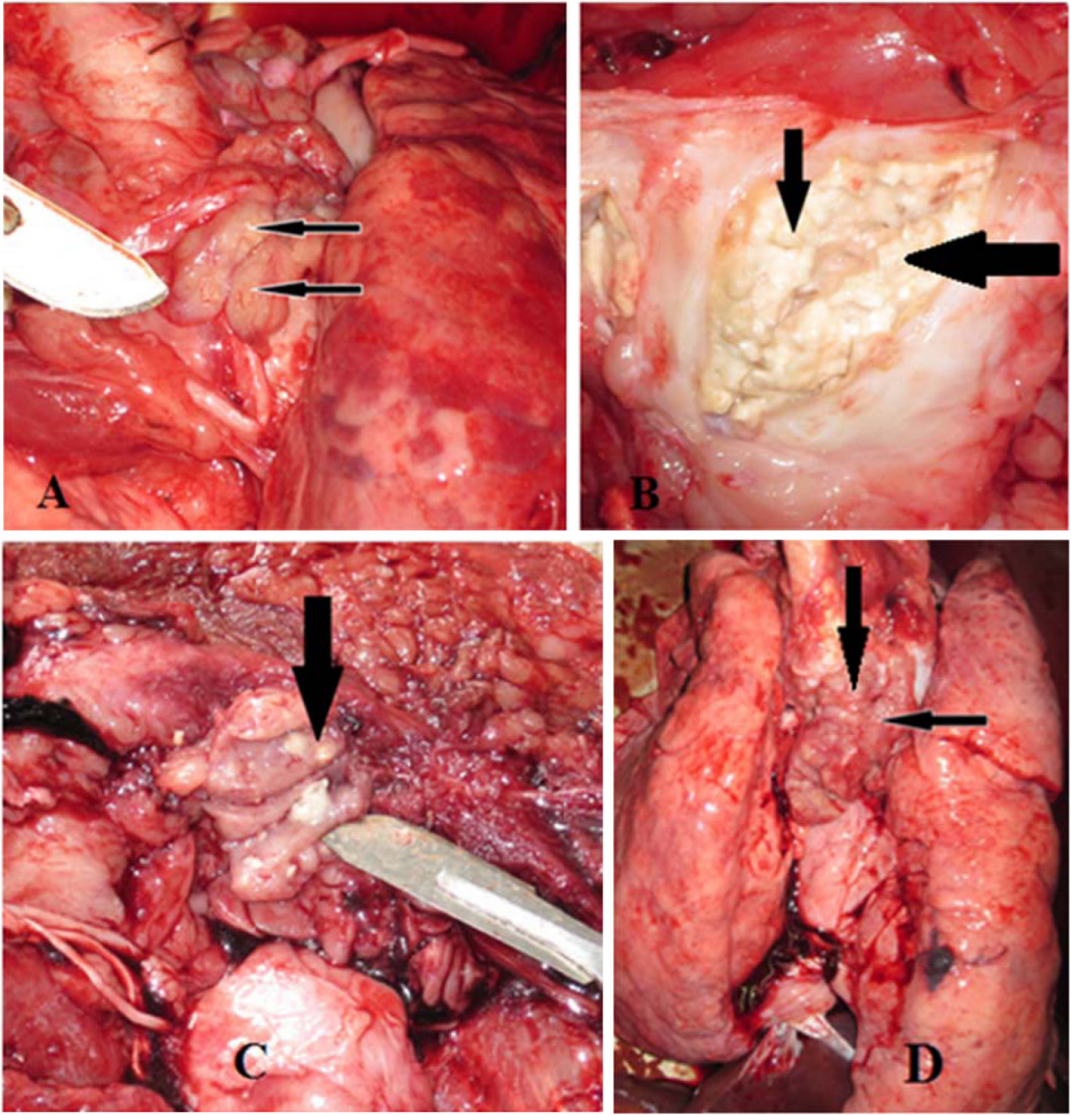
**Tuberculous lesions in various tissues of pigs at slaughter.** Tuberculous lesions indicated by solid arrows in various lymph nodes of pigs at slaughter. Bronchial lymph node (**A**), submandibular lymph nodes (**B**, **C**) and enlarged mediastinal lymph node (**D**).

Detection of gross lesions suggestive of tuberculosis in any of the tissues was used to classify the tissue as having TB lesions (Figure 1). The prevalence of TB in pigs was 5.8% (49/841) on the basis of pathological lesions. It was significantly higher in older pigs (more than one years of age) than in younger (one year and less) pigs (10.8% versus 3.4%, χ^2^ = 18.85; *P*<0.001, Table 1).

**Table 1:** Univariate analysis of potential risk factors associated with the presence of gross TB lesions in slaughter pigs

**Variables**	**No. examined**	**No. positive (%)**	**95% CI for positive**	**χ**^**2**^	***P*-value**
**Slaughter location**				4.86	0.028
Bishoftu	313	11 (3.5)	2.3:4.7		
Addis Ababa	528	38 (7.2)	5.5:9.0		
**Sex**				0.26	0.608
Male	373	20 (5.4)	3.9:7.0		
Female	468	29 (6.2)	4.6:7.8		
**Age**				18.85	< 0.001
≤ 1year	564	19 (3.4)	2.2:4.6		
>1 year	277	30 (10.8)	8.7:12.9		
**Origin**				27.67	< 0.001
Bishoftu	501	16 (3.2)	2.0:4.4		
Adama and Mojo	114	4 (3.5)	2.3:4.7		
Addis Ababa and Special Oromia Zone	226	29 (12.8)	10.5:15.1		
**Feed source**				5.37	0.021
Commercial mixed feed	740	38 (5.1)	3.6:6.6		
Grazing plus swill, offal and/or roaming	101	11 (10.9)	8.8:13.0		
**Water source**				0.44	0.833
River water	21	1 (4.8)	3.4:6.2		
Tap water	820	48 (5.9)	4.3:7.5		
**Floor type**				0.33	0.565
Soil	70	3 (4.3)	2.9:5.7		
Concrete	771	46 (6.0)	4.4:7.6		

No. examined: the number of pigs examined for gross TB lesion/s.

No. positive: the number of carcass with gross TB lesion/s.

Furthermore, the origin of the pigs was significantly associated with the prevalence (χ^2^ = 27.67; *P*< 0.001, Table 1). Pigs from Addis Ababa and the nearby Special Oromia Zone had the highest prevalence of 12.8%. Similarly, the prevalence was affected by the feeding practice (*P*<0.05) and location of the slaughterhouse (*P*<0.05). Pigs that were kept free grazing and fed with one or more of swill, poultry offal or left to roam for garbage had TB suggestive gross lesion/s more frequently than those fed on commercial mixed feed. In contrast, the variation in the prevalence between sex, floor type and water source was not statistically significant (*P*>0.05, Table 1).

Age and origin of animals were good indicators of TB prevalence in slaughtered pigs in the study areas (Table 2). Older pigs were more than twice (OR=2.55, CI = 1.36:4.78; *P=*0.004) as likely to present gross TB lesions than pigs less than one year of age. Likewise, origin of pigs significantly affected the prevalence of TB. Pigs from Addis Ababa and neighbouring Special Oromia Zone were more than three times (OR=3.29, CI=1.69:6.41; *P*<0.001) likely to show tuberculous lesion/s at slaughter than pigs from Bishoftu.

**Table 2:** Multivariate analysis of potential risk factors associated with the presence of gross TB lesions in slaughter pigs

**Variables**	**OR**	**95% CI for OR**		***P*-value**
		**Lower**	**Upper**	
**Age**				
≤ 1 year				0.003
>1 year	2.55	1.34	4.78	0.004
**Origin**				
Bishoftu				0.001
Adama and Mojo	0.87	0.28	2.70	0.812
Addis Ababa and Special Oromia Zone	3.29	1.69	6.41	<0.001

### Gross TB-suggestive lesions and mycobacterial culture

Gross TB-suggestive lesions were collected from submandibular, mesenteric, mediastinal, and bronchial lymph nodes and from lung and liver tissues. Mycobacterial culture positivity on LJ media enriched with glycerol was observed in 30.6% (15/49) of the tissues examined. No growth was observed on LJ media enriched with pyruvate. Ten of these isolates were AFB positive on Ziehl–Neelsen staining. Generally lesions were more frequently detected in lymph nodes and tissues of digestive tract than that of the respiratory tract. The percentage of gross TB lesions was highest in submandibular lymph nodes (28%) and lowest in mediastinal lymph nodes (7%). Culture positivity was observed on samples from mesenteric (20%) and submandibular (40%) lymph nodes and the lungs (40%).

### Molecular characterization of isolates

#### Identification of the genus *Mycobacterium*

**Figure 2:**
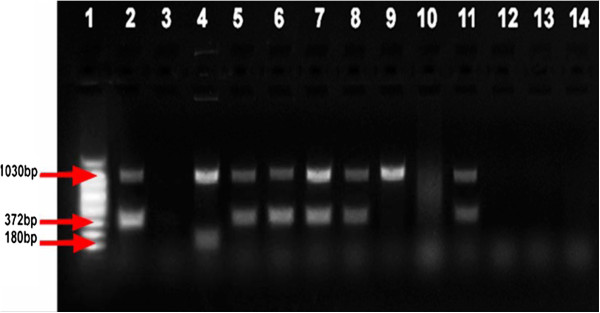
**Gel electrophoresis separation of PCR products of multiplex PCR genus typing on mycobacteria isolates from pigs.** Lane 1:100 bp DNA Ladder; Lane 2: *M. tuberculosis* H37Rv (positive control); Lane 3: Qiagen H_2_O (negative control); Lane 4: *M. avium* (positive control); Lanes 5–14 sample isolates from pigs.

Ten heat-killed AFB positive isolates were investigated for *Mycobacterium* genus typing (Figure 2). Six isolates (Lane 5, 6, 7, 8, 9 and 11) were confirmed to belong to the Genus *Mycobacterium* as they had a band size of 1030 bp which is specific to this genus. Five of these showed additional band size of 372 bp which is indicative of the MTC while the other isolate (Lane 9) did not produce this band. The other four isolates produced no band at all.

#### Speciation of *Mycobacterium tuberculosis* complex isolates

The five MTC isolates were then subjected to RD4 deletion and RD9 deletion typing (picture not shown) using the primers indicated in the methods section. A PCR product sizes of 335 bp (for RD4) and 396 bp (for RD9) were observed in all the isolates and thus confirming that they are *M. tuberculosis.*

#### Characterization of *Mycobacterium tuberculosis* isolates

**Figure 3:**
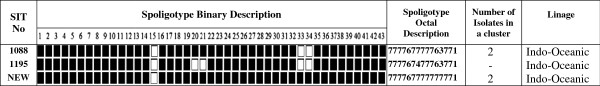
**Diagrammatic representation of spoligotype patterns of three clusters of five isolates of *M. tuberculosis *from five pigs slaughtered in central Ethiopia.** The five *M. tuberculosis* isolates were obtained from TB-compatible tissue lesions of slaughter pigs. These five isolates showed three distinct spoligotype patterns. They were SIT1088 (2 isolates), SIT1195 (one isolate), while two isolates (New) presented identical patterns which had not been reported to the soligotype database before. SIT = Spoligo-International-Typing number according to the SpolDB4/ SITVIT databases [26,27].

Characterization of the *M. tuberculosis* isolates was carried by spoligotyping. Three spoligotypes, two clusters with two isolates each one (one of them is new) and the last one with unique pattern with only one isolate were detected. One of the clusters is classified into SIT-1088 and the last one into SIT-1995 according to the Spoligo-International-Typing database [26,27] while the other cluster had not been previously described in the database (Figure 3).

## Discussion

In the present study, the occurrence of TB was investigated in slaughter pigs in two abattoirs from Addis Ababa and Bishoftu. Although the pig industry is growing in recent years and many piggeries are being established in many parts of the country public slaughtering is carried out only in Addis Ababa Abattoirs Enterprise slaughterhouse. The meat inspection activity is under close supervision by the federal ministry of agriculture where every carcass is inspected by recognized meat inspector. The slaughterhouse in Bishoftu is part of a private swine farm where small numbers of pigs are slaughtered. Carcass is not inspected by meat inspectors in this slaughterhouse. In addition to these two abattoirs are several backyard slaughters in many of small scale swine production systems which are favoured by increasing demand for pork by foreigners particularly by Chinese people dwelling in this regions.

This study is the first of its kind in Ethiopia to investigate the molecular epidemiology of TB in pigs. *Post-mortem* and bacteriological examinations as well as molecular typing were applied. The estimated prevalence of TB was approximately 6%, and is was lower than prevalence estimates reported from other African countries including Cameroon [28], Egypt [3] and Uganda [29]. On the other hand, it is higher than those reported from developed countries including New Zealand [4], The Netherlands [17], Czech Republic [12] and USA [30]. Such differences in the prevalence of TB in pigs between developing and developed countries could be associated with the eradication of TB in human and cattle in the developed world, where cattle served as sources of infection to pigs [30,31].

A significant association was detected among the age classes and the prevalence of TB lesion in pigs. Tuberculous lesions occurred more frequently in older pigs than younger pigs, which may be due to chronic nature of the disease, which requires a longer time to produce detectable lesions, and due to a longer period of exposure in older pigs. This findings is consistent with reports from other countries and in other species of animals [29,32,33].

Pigs that were kept on free grazing and fed with swill, offal or left to roam for garbage were found to harbour mycobacterial infections twice more frequently than those fed on commercial mixed feed, although the difference was not found to be significant. Outbreaks of *M. tuberculosis* in pigs which have been associated with the feeding of uncooked garbage from hospitals or residences housing human cases have been reported [30].

A higher estimated prevalence was recorded in pigs from Addis Ababa and Special Oromia Zone as compared to those slaughtered at Bishoftu. This variation could be a reflection of the husbandry system in that pigs are reared in Addis Ababa and the nearby Special Oromia Zone, where animals are kept under poor husbandry practices such as feeding swill, poultry litter, abattoir offal and garbage; sheltering with other domestic animals and confinement in poor housing system and where they have close contact with other animals and humans. In Ethiopia, the traditional small scale system, which is the predominant pig production system particularly in Addis Ababa and its nearby Special Oromia Zone, is characterised by absence or minimal health care, supplementary feeding and proper housing. It has been reported that the pig production is aggregated in the central part of the country [34], which could also promote the transmission of mycobacterial infections among the different farms through the movement of pigs from one farm to another particularly during establishment of new farms.

Lesions suggestive of TB were observed in the lungs, liver, head and were associated with mesenteric lymph nodes. Lesions appeared more frequently in the head, the mesenteric lymph nodes and in the liver. The presence of tuberculous lesions in the liver, the lymph nodes of the head and mesenteries may indicate infection by ingestion of contaminated feed, offal or infection from scavenging on contaminated garbage [30,32], while lesions in the lung and its associated lymph nodes suggest airborne transmission [35]. The observation of a higher proportion (>65%) of lesions in the lymph nodes of the head and gastrointestinal organs suggests ingestion to be the most common route of transmission in pigs. Similar observations were previously reported from Uganda [29,30]. However, this cannot exclude airborne transmission in pigs.

Only about 31% of the lesions yielded mycobacterial growth in culture. Lower rate of mycobacterial growth from tissues with gross TB lesions have been reported from Czech Republic [12], Egypt [3], Uganda [29] and many other countries [30]. Failure to demonstrate tubercle bacilli may be due to the occurrence of healed processes that contain no longer viable tubercle bacilli, or to microorganisms other than tubercle bacilli causing the lesions, such as *Rhodococcus equi* or *R. sputi* or inadequacy of the methods used for isolating tubercle bacilli [30,32]. It could also be due to subjective differences in identifying tuberculous lesions [20]. Nevertheless, culturing tubercle bacilli from such lesions needs further investigation so as to enable a maximum yield of viable organisms from culture for a correct interpretation of the results and thereby to appropriately explain the magnitude of the disease.

Isolation of *M. tuberculosis,* which is a predominant agent of tuberculosis in humans [1], from pigs suggests transmission between human and pigs, which could occur as a result of close contact between the two species [3], feeding of undercooked garbage or by sputum or body secretions from infected individuals [30]. The isolation of *M. tuberculosis* in the lung and its associated lymph nodes further supports the idea proposed by Parra and co-workers [36] that swine are not dead end hosts for mammalian tuberculosis. Previous studies suggested interspecies transmission of mycobacteria in Ethiopia [19,20,37-40]. Similar findings were also reported from other countries where the burden of TB in humans and animals is high and where transmission between these two is likely to occur on a regular basis [3,41]. Nonetheless, most reports from developed countries showed that lesions of TB in swine are due to members of the MAC [17,30,42-44]. In view of the fact that such reports are from countries, where there is an on**-**going mammalian TB control program, this difference may be due to the control of TB achieved by these countries [42].

Early in the 20^th^ century, when TB in cattle and humans was more prevalent, TB in swine was either due to *M. bovis* or *M. tuberculosis*. However, for example in the USA the avian type TB began to occur more frequently in swine by 1925. Today, isolation of mycobacteria other than *M. avium* from swine is uncommon in the USA. In rare cases, it occurs in pigs which are kept on the same premises with *M. bovis* infected cattle or which have been in close contact with *M. tuberculosis* infected humans [42]. After the enforcement of strict regulations for a test-and-slaughter policy, the prevalence of bovine type of TB in swine gradually declined concurrently with the eradication of the disease in cattle in most western countries [17,30,31].

In this study, one of the AFB positive isolates had a PCR product size indicative for the Genus *Mycobacterium* but none for MTC or MAC upon genus typing using multiplex PCR. The isolate was hence assumed to be a member of the mycobacteria other than tuberculosis (MOTT). Isolation of MOTT from swine has previously been reported from other countries [3,4]. In Ethiopia, MOTT have been isolated from cattle [20] and camels [33] with tuberculous-like lesions, which indicates their involvement in a broad range of animal species [45] and their role as a cause of tuberculosis.

The two isolates in one of the clusters with identified SIT1088 have been detected in Egypt [46], India, South Africa and Portugal, while the other single isolate identified as SIT1995 has been reported from India [27]. In Ethiopia, several studies recently reported new strains of *M. tuberculosis*[20,33,40,47].

## Conclusions

The isolation of *M. tuberculosis* from pigs suggests a possible risk of interspecies transmission particularly between pigs and humans. With this suggestion in mind, and with the expected rapid expansion of swine production within a couple of decades, it is likely that swine may play a role in increasing the incidence of TB in the country. Hence, establishing feasible control methods is recommended.

## Competing interests

The authors declare that they have no competing interests.

## Authors’ contributions

All authors participated in the design of the study, GA and SMA conceived of the study, carried out the culturing and molecular typing, performed the statistical analysis and drafted the manuscript. SMA collected samples and epidemiological data. All authors reviewed and approved the final manuscript.
